# Differential associations of subcutaneous and visceral fat with bone turnover markers: A study on bariatric surgery patients with severe obesity and individuals without obesity

**DOI:** 10.1038/s41366-025-01888-1

**Published:** 2025-08-26

**Authors:** Prince Dadson, Eleni Rebelos, Maria K. Jaakkola, Milena Monfort-Pires, Ronja Ojala, Henri Honka, Kari K. Kalliokoski, Riku Klén, Pirjo Nuutila, Kaisa K. Ivaska

**Affiliations:** 1 Turku PET Centre, University of Turku, Turku, Finland; 2 Turku PET Centre, Turku University Hospital, Turku, Finland; 3 InFLAMES Research Flagship Center, University of Turku, Turku, Finland; 4 Institute of Biomedicine, University of Turku, Turku, Finland

**Keywords:** Metabolic bone disease, Weight management, Obesity, Fat metabolism

## Abstract

**Background:**

Obesity suppresses bone turnover markers (BTMs) in circulation, and weight loss after metabolic and bariatric surgery (MBS) increases BTM levels. However, the relationship between regional fat distribution and BTMs has not been thoroughly investigated. This study aimed to determine which specific fat compartments - namely abdominal and femoral subcutaneous fat (SF), intraperitoneal fat, extraperitoneal fat, and total visceral fat (VF) - have the greatest impact on circulating BTM levels following weight loss induced by MBS.

**Methods:**

The study comprised a cohort of individuals with severe obesity (*n* = 46) studied before and 6 months after MBS, either sleeve gastrectomy (SG, *n* = 25) or Roux-en-Y gastric bypass (RYGB, *n* = 21). Healthy individuals without obesity (*n* = 25) served as controls. Regional fat depots were quantified with magnetic resonance imaging. The BTMs included Tartrate-Resistant Acid Phosphatase 5b, C-terminal Telopeptide of Type I Collagen (CTX), Procollagen Type I N-terminal Propeptide (PINP), and Total (TotalOC), Carboxylated (cOC), and Undercarboxylated (ucOC) osteocalcin.

**Results:**

In the pooled baseline analysis, no significant associations were observed between fat depots and BTMs (all *p* > 0.05). Postoperatively, distinct patterns emerged between surgical groups. In the SG cohort, femoral SF was inversely associated with cOC levels (*p* < 0.05) compared to the RYGB group. Following RYGB, extraperitoneal, intraperitoneal, and total VF were significantly associated with TotalOC, while intraperitoneal and total VF were also negatively associated with ucOC (all *p* < 0.05) compared to SG. All *p*-values were adjusted for false discovery rate to correct for multiple comparisons.

**Conclusions:**

The findings suggest a specific interaction between intraperitoneal, extraperitoneal, and total visceral compartments and bone metabolism following RYGB. These observed relationships highlight the need for clinicians to consider regional fat distribution when assessing bone health in post-MBS patients.

**ClinicalTrials.gov registration numbers:**

NCT00793143 and NCT01373892.

## Introduction

Obesity, characterized by excessive accumulation of fat in the body, is a significant global public health challenge [1]. Although obesity has been associated with improved bone strength and increased bone mineral density (BMD) in skeletal regions such as the lumbar spine and femoral neck, likely due to the anabolic effect of load-bearing effects of increased body weight on bones [2], there is growing evidence that obesity, especially severe obesity (BMI of 40 kg/m^2^ or greater), may be associated with increased risk of fractures [3]. This potential risk is thought to be mediated by various mechanisms, including alterations in bone-regulating hormones, inflammation, and bone cell metabolism [3]. The physiological and biochemical mechanisms underlying this obesity-bone paradox need to be further investigated.

It is increasingly recognized that it is not obesity per se, but rather the distribution of fat in localized regions that are closely linked to the decline in BMD and the increased risk for fragility fractures [4]. Specifically, a cross-sectional study found a negative correlation between visceral fat (VF) volume and trabecular BMD, while no significant association was observed with abdominal subcutaneous fat (SF) [5, 6]. Another study reported a negative association between SF mass and BMD, as well as a U-shaped relationship between VF and BMD [7]. Femoral SF mass is positively associated with a protective lipid and glycemic profile, which are established risk factors for cardiometabolic conditions [8]. This association suggests that individuals with higher levels of femoral SF mass may also experience potentially beneficial effects on BMD. However, there is limited understanding of the impact of femoral fat accumulation on overall bone metabolism.

Imaging and cadaver studies have shown that VF is not a homogeneous fat depot [9]. Instead, it can be divided into intraperitoneal and extraperitoneal fat compartments [4]. Intraperitoneal fat is primarily composed of mesenteric and omental fat, and it is thought to be most strongly associated with the metabolic health risks of obesity [10]. This association is largely attributed to the “portal hypothesis” which proposes that intraperitoneal fat drains directly into the portal vein, exposing the liver to high levels of free fatty acids and pro-inflammatory factors [11]. In contrast, extraperitoneal fat is located within the intrapelvic, preperitoneal, retroperitoneal regions [12]. It drains into the inferior vena cava, entering the systemic circulation while bypassing the liver [13]. So far, to our knowledge no studies have investigated the potential differential effects of intraperitoneal and extraperitoneal fat on bone metabolism.

Bone turnover markers (BTMs) are biochemical surrogate markers of the activities of osteoblasts and osteoclasts, providing an estimate of bone metabolism at a whole-body level [14]. BTMs are useful tools in the assessment of response to bone-active medication and they may assist fracture risk prediction [15] and provide supplementary information to radiographic measures of bone mass [16]. Individuals with type 2 diabetes mellitus (T2DM) exhibit suppressed bone turnover and lower circulating BTM values compared to those without diabetes [17]. This reduction is further exacerbated in those with T2DM who also have metabolic syndrome and increased VF mass [18].

The aim of the present study was to evaluate potential differences in the association patterns between various fat depot volumes assessed (abdominal and femoral SF, as well as intraperitoneal, extraperitoneal, and total VF) and BTMs. Associations were analysed in individuals with severe obesity before and after metabolic and bariatric surgery (MBS), as well as in age-matched controls, to explore the depot-specific role of fat in bone metabolism. This study builds upon our previous findings on the effects of MBS-induced weight loss on BTMs, namely C-terminal telopeptide of type I collagen, procollagen type I N-terminal propeptide, tartrate-resistant acid phosphatase isoform 5a/b, undercarboxylated osteocalcin, and total osteocalcin [19] by including an analysis of region-specific fat depots, which have not been previously studied in this context.

## Methods

### Human participants, study design and methods

A total of 46 participants (female/male, 42/4) were recruited from individuals undergoing MBS at the Hospital District of Southwest Finland [20]. In brief, inclusion criteria included an age range of 18–60 years and a BMI of ≥ 40 kg/m² (or ≥ 35 kg/m² with an additional obesity-related comorbidity). Among the 46 participants with severe obesity, 18 had T2DM, and out of the 28 participants without T2DM, 11 had impaired fasting glucose (IFG) or impaired glucose tolerance (IGT) [21]. Twenty-five (female/male, 23/2) individuals without obesity served as controls. The participants had no known metabolic bone disease (except for one hyperparathyroidism) and none were taking antiresorptive or bone-anabolic medication. The protocols were approved by the Ethics Committee of the Wellbeing Services County of Southwest Finland and were conducted in compliance with the Code of Ethics of the World Medical Association (Helsinki Declaration). Written informed consent was obtained prior to the studies. Trial registrations numbers: NCT00793143 and NCT01373892.

### Analysis of biochemical and inflammatory markers

Plasma glucose, glycated hemoglobin, serum insulin, and high-sensitivity C-reactive protein were measured as described previously [21]. Glycemic indices were assessed using a standard 75 g oral glucose tolerance test (OGTT) [22]. Furthermore, insulin resistance was evaluated using two methods: the Homeostasis Model Assessment of Insulin Resistance (HOMA-IR) using the formula [(fasting glucose × fasting insulin)/22.5] [23] and the Matsuda Index [24]. Stored serum samples were analyzed for cytokine and adipokine levels using the Bio-Plex 200 system (Bio-Rad Laboratories, Inc., CA, USA) and Bio-Plex Manager Software version 4.1. Multiplex quantification of cytokines was performed using the MILLIPLEX MAP Kit Human Adipokine Magnetic Bead Panels 1 and 2 (Millipore Corporation, MA, USA).

### Bone turnover markers

Fasting morning serum samples were collected before and 6 months after MBS, stored at -80 °C, and used for BTM analysis, as previously described [19]. Bone resorption was assessed using C-terminal crosslinked telopeptides of type I collagen [CTX, (CrossLaps®, IDS Ltd., UK)], osteoclast activity with osteoclast-specific tartrate-resistant acid phosphatase 5b [TRAcP5b (BoneTRAP® ELISA, IDS Ltd., UK)], and low-grade inflammation by measuring TRAcP5a [25]. Bone formation was evaluated using N-terminal propeptide of type I collagen [PINP, (IDS Ltd., UK)]. Serum total osteocalcin (TotalOC) and carboxylated (cOC) were determined with a two-site immunoassay, while undercarboxylated osteocalcin (ucOC) was assessed with an in-house assay based on hydroxyapatite binding [19]. The ‘Resorption index’ (CTX/TRAcP5b) and ‘Coupling index’ (PINP/CTX) were calculated to assess bone remodeling balance.

### Measurement of fat mass

**Fig. 1:**
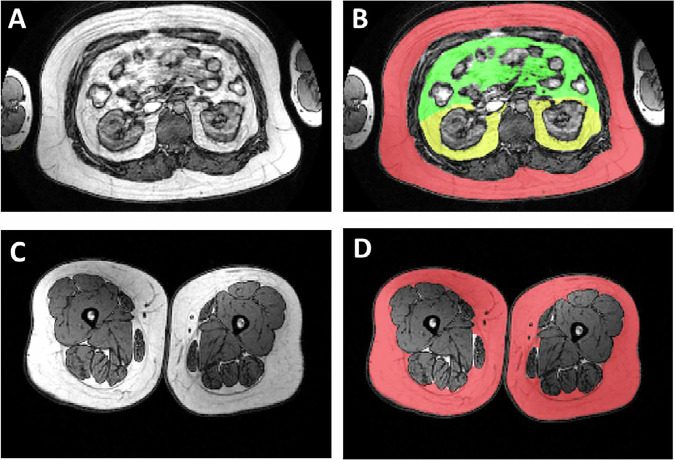
Regional fat distribution for the study participants. The upper panel, Figs. 1**A** and 1**B**, illustrate abdominal fat compartment segmentation with subcutaneous (RED), intraperitoneal fat (GREEN), and extraperitoneal fat (YELLOW). The lower panel, Figs. 1**C** and 1**D**, shows femoral subcutaneous fat (RED).

Body fat content was measured using bioelectric impedance (Omron BF400). Volumes of different fat depots were measured using a 1.5 Tesla MRI (Tesla Intera system; Philips Medical Systems, Best, the Netherlands). Axial T1-weighted dual fast field echo images (echo time 2.3 and 4.6, repetition time 120 ms, slice thickness 10 mm without gap) were acquired for the abdominal and femoral regions. Femoral SF mass was calculated from the femoral head to the patella surface. The abdominal visceral region was separated into the intraperitoneal and extraperitoneal compartments using specific anatomical reference points (Fig. 1) [26]. The fat regions were analyzed using sliceOmatic (Tomovision, Montreal, Quebec, Canada). Fat volumes (cm³) were converted to mass (kg) by taking into account the tissue density [27].

### Metabolic and bariatric surgery

The participants with severe obesity followed a 4-week very-low-calorie (VLCD) diet (800 kcal/day) before undergoing MBS. The individuals with severe obesity underwent either RYGB (*n* = 21) or sleeve gastrectomy (SG, *n* = 25). Post-MBS studies were conducted 6 months after the surgical intervention [21, 27, 28]. All subjects were instructed to take daily calcium (1000 mg) and vitamin D3 (20 μg) supplements and multivitamin tablet (including 10 μg vitamin D3) after the surgery [19].

### Statistical analysis

The study presented continuous variables as means with standard deviations. Normality of distribution was assessed using the Shapiro-Wilk test. Variables, including all BTMs, that were not normally distributed underwent logarithmic transformation before analysis. Unpaired t-tests were used to compare means between individuals with severe obesity and controls individuals without obesity for metabolic, clinical, and bone turnover marker variables. Paired t-tests were used to assess changes in these variables before and after metabolic bariatric surgery.

We used mixed-effects models to evaluate the impact of abdominal SF, extra- and intraperitoneal VF, total VF, and femoral SF on various bone markers (TRACP5a, TRACP5b, CTX, PINP, TotalOC, cOC, ucOC). Besides the fat compartments, surgery type, age, weight, and interaction between the fat compartment and the surgery type were used as fixed covariates to explain bone marker levels while participant identifier was used as a random effect. Control samples, lacking defined surgery types, were excluded from the model fitting. A conventional false discovery rate (FDR) cutoff of 0.05 signified statistical significance. Correlations between BTMs, with the different fat depots, inflammatory and glycemic parameters were assessed using Spearman’s rank correlation coefficient. Analyses were performed using R packages lmerTest (version 3.1-3) and lme4 (version 1.1-34), and Statistical Package for the Social Sciences (SPSS, Version 29.0 IBM Corp., Armonk, NY) was used for visualization of the data.

## Results

**Fig. 2:**
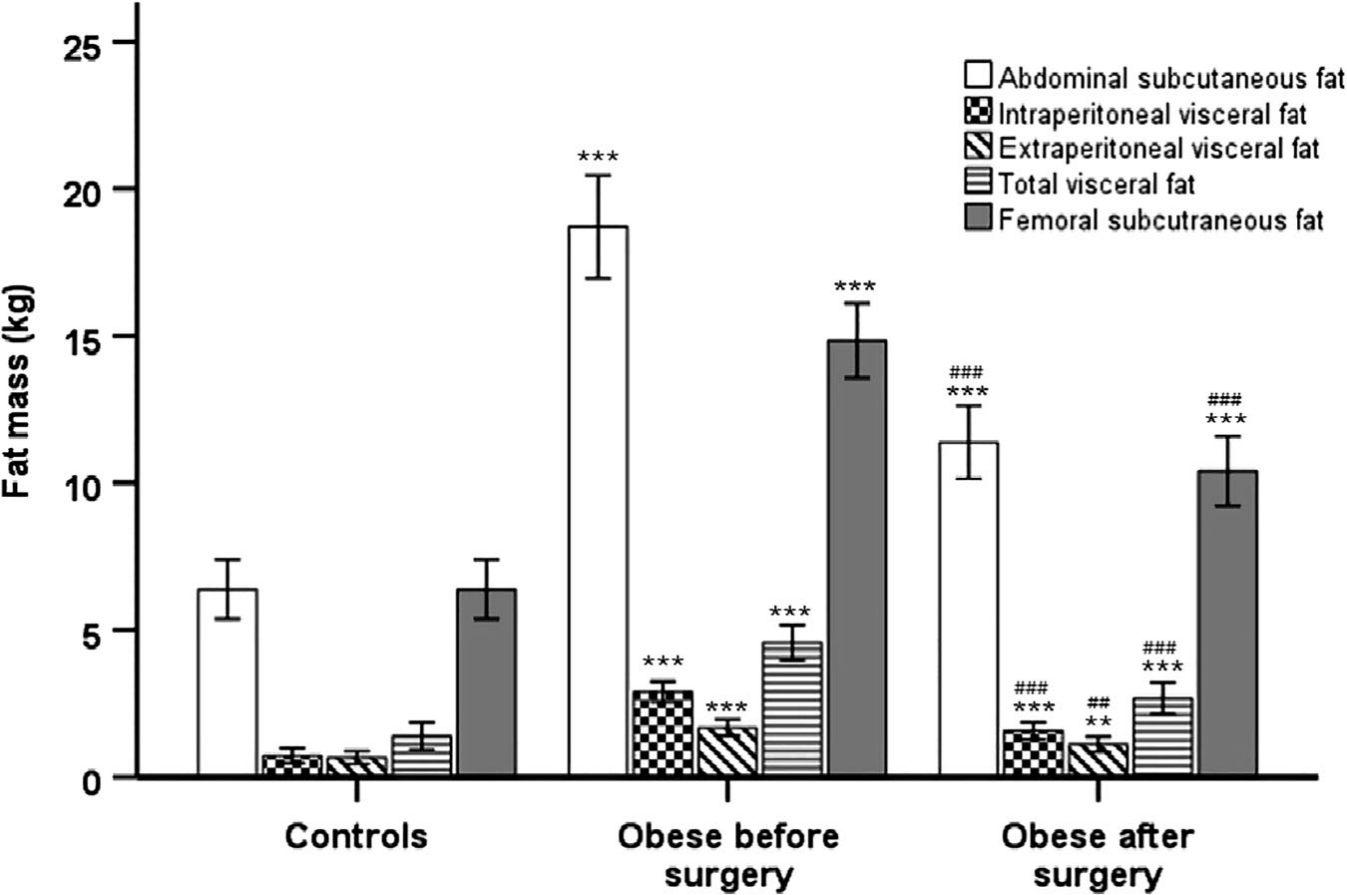
Fat mass values of individuals with severe obesity before and after weight loss following metabolic bariatric surgery, compared to healthy individuals without obesity. Statistical significance is indicated as follows: *p < 0.05, **p < 0.01, ***p < 0.001 for independent samples t-tests comparing obese vs. controls; ^#^*p* < 0.05, ^# #^*p* < 0.01, ^# # #^*p* < 0.001 for paired samples t-test comparing individual with severe obesity before vs. after surgery. Error bars represent the 95% confidence interval.

The descriptive characteristics of the study participants are shown in Table 1. and previously published reports [19, 21]. As expected, all indices of adiposity including of abdominal and femoral SF, as well as intra- and extraperitoneal VF were found to be significantly higher in the individuals with severe obesity compared to the healthy lean control subjects (Fig. 2, all *p* < 0.001) [27, 28]. Moreover, persons with severe obesity exhibited poorer glycemic and lipid profiles and were characterized by low-grade inflammation when compared to the controls, as presented in Table 1 and reported in previous studies [19, 21].

**Table 1:** Anthropometric and metabolic characteristics of the study subjects.

Variables	Controls (*n* = 25)	Severely obese (*n* = 46)		^#^*P*-value
		Before surgery	After surgery	
Age (years)	45.84 ± 10.23	44.93 ± 9.51	45.22 ± 9.34	0.888
Sex (Female/male)	23/2	42/4	42/4	–
Weight (kg)	64.79 ± 7.74	116.69 ± 13.79***	90.04 ± 13.47***	<0.001
BMI (kg/m²)	23.04 ± 2.5	42.11 ± 4.03***	32.51 ± 4.17***	0.939
Waist (cm)	77.38 ± 9.4	119.13 ± 10.3***	100.33 ± 10.93***	<0.001
Body fat (%)	31.13 ± 6.54	49.3 ± 5.81***	42.42 ± 5.41***	<0.001
Abdominal SF mass (kg)	4.08 ± 1.74	18.7 ± 5.81***	11.38 ± 4.08***	<0.001
Intraperitoneal VF mass (kg)	0.73 ± 0.59	2.89 ± 1.19***	1.57 ± 0.95***	<0.001
Extraperitoneal VF mass (kg)	0.66 ± 0.5	1.68 ± 0.93***	1.11 ± 0.85**	<0.001
Total VF mass (kg)	1.39 ± 1.08	4.57 ± 2.0***	2.68 ± 1.75***	<0.001
Femoral SF mass (kg)	6.38 ± 2.28	14.83 ± 4.23***	10.4 ± 3.88***	<0.001
Fasting glucose (mmol/L)	5.36 ± 0.55	6.32 ± 1.23***	5.39 ± 0.65	<0.001
2-h glucose (mmol/L)	5.63 ± 1.18	8.87 ± 3.21***	5.81 ± 2.82	<0.001
Fasting insulin (mmol/L)	6.0 ± 3.55	15.34 ± 11.4***	7.53 ± 4.73	<0.001
Cholesterol (mmol/L)	4.68 ± 0.85	4.29 ± 0.81	4.19 ± 0.74*	0.549
Triglycerides (mmol/L)	0.71 ± 0.31	1.25 ± 0.45***	1.0 ± 0.42**	0.005
HDL-cholesterol (mmol/L)	1.86 ± 0.44	1.26 ± 0.24***	1.43 ± 0.27***	0.008
LDL-cholesterol (mmol/L)	2.5 ± 0.7	2.46 ± 0.71	2.3 ± 0.66	0.135
HbA1c (%)	5.64 ± 0.28	5.7 ± 0.62	5.52 ± 0.39	0.009
CRP (mg/L)	0.88 ± 0.90	4.28 ± 3.98***	1.58 ± 1.55*	<0.001
Matsuda Index	8.9 ± 5.68	3.19 ± 1.96***	5.09 ± 2.47***	0.007
HOMA-IR	1.42 ± 0.93	4.7 ± 5.41***	1.83 ± 1.24*	<0.001
IL-6 (pg/mL)	2.65 ± 2.87	3.16 ± 2.26*	2.03 ± 1.39	<0.001
IL-8 (pg/mL)	4.78 ± 2.04	6.14 ± 2.91*	6.98 ± 6.13	0.297
TNF-α (pg/mL)	4.14 ± 2.6	6.05 ± 3.42*	5.81 ± 3.31*	0.567
MCP-1 (pg/mL)	235.23 ± 117.67	309.6 ± 163.47*	293.39 ± 125.95*	0.789
Leptin (ng/mL)	9.09 ± 6.68	47.76 ± 20.45***	23.22 ± 15.95***	<0.001
Resistin (ng/mL)	14.02 ± 4.52	17.45 ± 5.57**	17.7 ± 5.67**	0.617
Adiponectin (μg/mL)	19.71 ± 11.8	21.36 ± 48.77	20.76 ± 12.66	<0.001

Data are presented as the mean ± SD. *SF* subcutaneous fat, *VF* visceral fat, *2-h glucose* glucose concentration two hours after a standardized 75-gram oral glucose tolerance test, *HDL* high-density lipoprotein, *LDL* low-density lipoprotein cholesterol, *HbA1c* hemoglobin A1c, *CRP* C-reactive protein, *HOMA-IR* homeostatic model assessment of insulin resistance; interleukin-6/8 (IL-6/8), *TNF-α* tumor necrosis factor-alpha; MCP-1, monocyte chemoattractant protein-1. *p < 0.05, **p < 0.01, ***p < 0.001 for independent samples *t*-tests comparing individuals with severe obesity vs. controls subjects without obesity; #*p*-value for paired samples t-test comparing individuals with severe obesity before vs. after metabolic and bariatric surgery; *p* < 0.05 was considered statistically significant.

The levels of BTMs have been previously reported in this same cohort, however this previous study categorized the individuals with severe obesity into those with and without T2DM before surgery, with a focus on metabolic health and T2DM remission [19]. For this study, weight loss induced by MBS led to an increase in both bone resorption markers (TRACP5b, CTX) and bone formation markers (PINP, TotalOC), as shown in Table 2 and previously published findings [19].

**Table 2:** Bone turnover markers and other bone variables in the study participants.

Variables	Controls (*n* = 25)	Severely obese (*n* = 46)		^#^*P*-value
		Before surgery	After surgery	
TRACP5a (U/L)	2.17 ± 0.58	2.21 ± 0.5	2.51 ± 0.6*	0.007
TRACP5b (U/L)	2.58 ± 0.94	2.44 ± 0.64	3.63 ± 1.03***	<0.001
CTX (ng/mL)	0.52 ± 0.37	0.32 ± 0.18**	0.8 ± 0.37***	<0.001
PINP (ng/mL)	54.07 ± 39.72	38.27 ± 15.1*	73.12 ± 29.32***	<0.001
TotalOC (ng/mL)	8.39 ± 3.69	5.25 ± 1.92***	9.63 ± 3.86	<0.001
cOC (ng/mL)	7.79 ± 3.83	4.94 ± 2.41**	8.44 ± 3.97	<0.001
ucOC (ng/mL)	2.37 ± 1.91	1.71 ± 1.09	4.24 ± 2.7**	<0.001
TRACP5a/b (ratio)	0.94 ± 0.38	0.93 ± 0.19	0.72 ± 0.17*	<0.001
cOC/TotalOC (ratio)	0.93 ± 0.14	0.92 ± 0.28	0.87 ± 0.22	0.243
ucOC/TotalOC (ratio)	0.26 ± 0.16	0.3 ± 0.17	0.43 ± 0.17***	<0.001
PINP/CTX (ratio)	112.05 ± 32.69	142.57 ± 58.4*	96.58 ± 28.3*	<0.001
CTX/TRACP5b (ratio)	0.19 ± 0.08	0.13 ± 0.07***	0.22 ± 0.08	<0.001
Ca^2+^(mmol/L)	1.24 ± 0.03	1.23 ± 0.04	1.23 ± 0.05	0.253
Phosphate (mmol/L)	1.03 ± 0.13	0.98 ± 0.15	1.09 ± 0.16	<0.001

Data are presented as the Mean ± SD. *TRACP5a* Tartrate-Resistant Acid Phosphatase Isoform 5a, *TRACP5b* Tartrate-Resistant Acid Phosphatase Isoform 5b, *CTX* C-Terminal Telopeptide of Type I Collagen, *PINP* N-Terminal Propeptide of Type I Collagen, *TotalOC* Total Osteocalcin, *cOC* Carboxylated Osteocalcin, *ucOC* Undercarboxylated Osteocalcin, *TRACP5a/b* Ratio of Tartrate-Resistant Acid Phosphatase Isoforms 5a to 5b, *cOC/TotalOC* Ratio of Carboxylated Osteocalcin to Total Osteocalcin, *ucOC/TotalOC* Ratio of Undercarboxylated Osteocalcin to Total Osteocalcin, *PINP/CTX* Ratio of N-Terminal Propeptide of Type I Collagen to C-Terminal Telopeptide of Type I Collagen, *CTX/TRACP5b* Ratio of C-Terminal Telopeptide of Type I Collagen to Tartrate-Resistant Acid Phosphatase Isoform 5b; Ca^2+^: Concentration of ionized calcium in the blood serum. *p < 0.05, **p < 0.01, ***p < 0.001 for the Mann-Whitney U test comparing individuals with severe obesity vs. controls subjects without obesity; #*p*-value for the Wilcoxon Signed-Rank test comparing individuals with severe obesity before vs. after metabolic bariatric surgery. *p* < 0.05 was considered statistically significant.

**Fig. 3:**
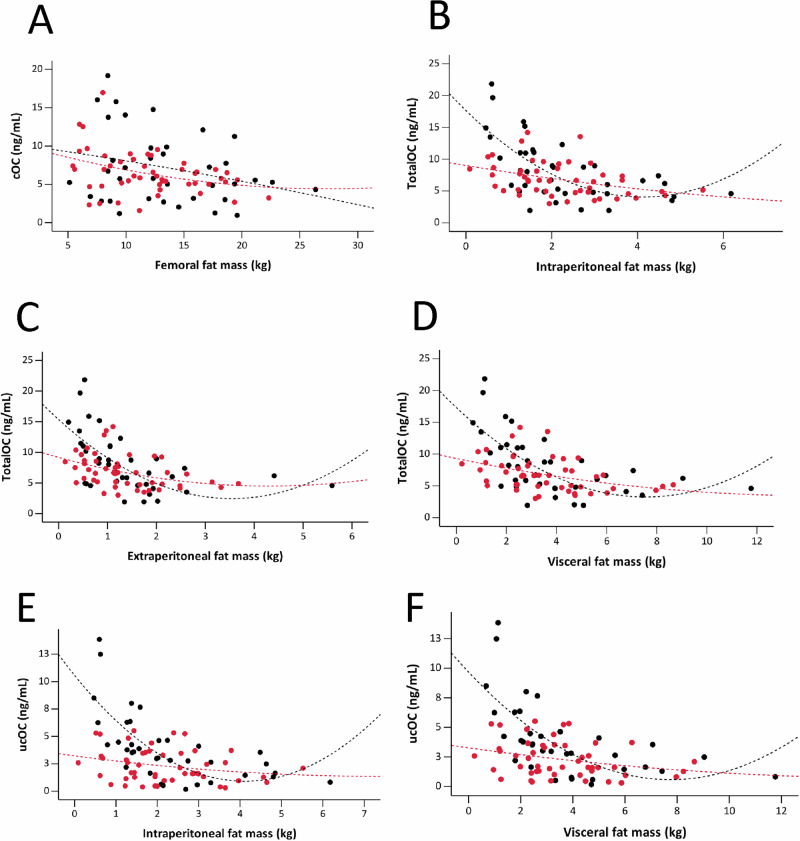
Scatter plots showing the differential associations between regional fat mass and bone turnover markers by type of metabolic and bariatric surgery using the fitted mixed-effects model. Femoral subcutaneous fat showed a negative association with carboxylated osteocalcin (cOC) [**A**] (*r* = -0.362, *p* = 0.047). For the Roux-en-Y gastric bypass group, intraperitoneal [**B**] (*r* = -2.116, *p* = 0.011), extraperitoneal [**C**] (*r* = -1.947, *p* = 0.029), and total visceral fat [**D**] (*r* = -1.066, *p* = 0.013) were negatively associated with total osteocalcin (TotalOC). Intraperitoneal [**E**] (*r* = -1.560, *p* = 0.011) and total visceral fat [**F**] (*r* = -0.712, *p* = 0.029) were negatively associated with undercarboxylated osteocalcin (ucOC). Points and lines of best fit are colored to differentiate between sleeve gastrectomy (BLACK) and Roux-en-Y gastric bypass (RED) metabolic and bariatric surgeries. Statistically significant correlations are based on false discovery rate (FDR) < 0.05.

In a pooled baseline data consisting of individuals with severe obesity and controls, there were no significant associations between depot fat mass and any of the BTMs (FDR-adjusted *p* > 0.05). Using mixed-effects models, fat compartments analysed alone and without the interaction terms showed no statistically significant associations (all FDR *p* < 0.05) with any BTMs (Table 3). However, the type of MBS had significant impact of the associations between regional fat distribution and BTMs. Following SG, a borderline significant negative association was found between femoral SF and cOC (Table 3, Fig. 3A). After RYGB, intraperitoneal (Table 3, Fig. 3B), extraperitoneal (Table 3, Fig. 3C), and total VF (Table 3, Fig. 3D) were significantly negatively associated with TotalOC. Additionally, intraperitoneal (Table 3, Fig. 3E) and total VF (Table 3, Fig. 3F) were negatively associated with ucOC.

**Table 3:** Mixed effects model of fat compartments predicting bone turnover markers.

	TRACP5a	TRACP5b	CTX	PINP	TotalOC	cOC	ucOC
**Fat alone**							
Abdominal SF	0.007 (0.810)	0.027 (0.554)	0.014 (0.430)	1.831 (0.109)	-0.013 (0.914)	0.027 (0.832)	0.023 (0.810)
Extraperitoneal VF	-0.013 (0.914)	-0.140 (0.560)	-0.067 (0.474)	-3.577 (0.574)	-0.684 (0.439)	-0.891 (0.386)	-0.337 (0.554)
Intraperitoneal VF	0.030 (0.810)	-0.077 (0.683)	-0.060 (0.430)	-1.609 (0.775)	-0.474 (0.528)	-0.739 (0.386)	-0.190 (0.641)
Total visceral fat	0.005 (0.916)	-0.061 (0.577)	-0.037 (0.430)	-1.448 (0.631)	-0.348 (0.430)	-0.503 (0.292)	-0.148 (0.560)
Femoral SF	-0.015 (0.724)	0.000 (0.996)	0.019 (0.430)	1.817 (0.341)	0.068 (0.761)	0.131 (0.554)	0.079 (0.574)
**Fat × sleeve gastrectomy**							
Abdominal SF	-0.011 (0.810)	-0.032 (0.586)	-0.017 (0.528)	-1.294 (0.536)	-0.058 (0.797)	-0.206 (0.382)	-0.078 (0.586)
Extraperitoneal VF	-0.259 (0.515)	0.041 (0.914)	0.020 (0.890)	-8.421 (0.560)	-0.820 (0.528)	-0.948 (0.430)	-0.506 (0.560)
Intraperitoneal VF	0.043 (0.846)	0.102 (0.795)	-0.029 (0.810)	-2.550 (0.810)	-0.511 (0.574)	-0.542 (0.560)	-0.353 (0.583)
Total visceral fat	-0.045 (0.795)	0.043 (0.816)	-0.005 (0.916)	-2.811 (0.657)	-0.340 (0.552)	-0.371 (0.528)	-0.225 (0.560)
Femoral SF	-0.039 (0.536)	-0.096 (0.202)	-0.025 (0.430)	-2.087 (0.430)	-0.215 (0.430)	**-0.362 (0.047)**	-0.147 (0.439)
**Fat × gastric bypass**							
Abdominal SF	0.017 (0.810)	0.037 (0.710)	-0.026 (0.536)	-0.895 (0.775)	-0.223 (0.554)	-0.058 (0.829)	-0.130 (0.584)
Extraperitoneal VF	-0.037 (0.847)	-0.187 (0.572)	-0.117 (0.430)	-5.390 (0.584)	**-1.974 (0.029)**	-1.355 (0.165)	-1.157 (0.115)
Intraperitoneal VF	-0.108 (0.616)	-0.233 (0.515)	-0.157 (0.165)	-8.831 (0.430)	**-2.116 (0.011)**	-1.440 (0.108)	**-1.560 (0.011)**
Total visceral fat	-0.040 (0.761)	-0.109 (0.542)	-0.070 (0.341)	-3.710 (0.528)	**-1.066 (0.013)**	-0.726 (0.109)	**-0.712 (0.029)**
Femoral SF	0.024 (0.696)	0.013 (0.846)	-0.020 (0.554)	-2.529 (0.430)	0.078 (0.797)	0.058 (0.810)	-0.092 (0.657)

Coefficients and the corresponding FDR values (in parentheses) of different fat compartments (rows) from the mixed-effect model explaining the bone marker levels (columns) using age, weight, surgery type, and the fat compartment as fixed effects and sample donor as a random effect. *SF* subcutaneous fat, *VF* visceral fat. *TRACP5a* Tartrate-Resistant Acid Phosphatase Isoform 5a, *TRACP5b* Tartrate-Resistant Acid Phosphatase Isoform 5b, *CTX* C-Terminal Telopeptide of Type I Collagen, *PINP* N-Terminal Propeptide of Type I Collagen, *TotalOC* Total Osteocalcin, *cOC* Carboxylated Osteocalcin, *ucOC* Undercarboxylated Osteocalcin. The values are rounded to three decimals, and the statistically significant ones (*p* < 0.05) are highlighted by bolding.

Our investigation into the associations between circulating cytokines and adipokines and BTMs revealed several significant correlations. Specifically, IL-8 and TNF-α displayed a positive correlation with TRACP5a, an isoform produced by inflammatory macrophages (Supplementary Table 1). MCP-1 was negatively correlated with cOC (Supplementary Table 1). Leptin levels exhibited negative correlations with several BTMs, namely CTX, TotalOC, and cOC (Supplementary Table 1, all *p* < 0.05).

Supplementary Fig. 1 presents additional analyses performed to assess the association between BTMs and glycemic parameters among the study participants. Our findings revealed negative correlations between the 2-h glucose level and CTX (Supplementary Fig. 1A), TotalOC (Supplementary Fig. 1B), PINP (Supplementary Fig. 1C), and ucOC (Supplementary Fig. 1D) (all FDR < 0.05).

## Discussion

It has previously been shown that while severe obesity suppresses bone remodeling, this remodeling process is reactivated following MBS [19]. Here, we elaborated further on the existing literature by assessing the effects of MBS on the associations of different fat depots with BTMs. Our main finding was that after SG, femoral SF was negatively associated with cOC a pattern not observed in the RYGB group. In contrast, after RYGB, the VF compartments were negatively associated with both TotalOC and ucOC, associations not seen following SG. While SG is solely restrictive, RYGB combines both restriction and malabsorption, leading to a more pronounced effect on fat and micronutrient absorption, and consequently, on bone health [29]. RYGB is also associated with a more pronounced reduction in BMD, a greater increase in BTMs, and a higher risk of fragility fractures compared to SG [30] even when patients received high doses of vitamin D and calcium supplements [31, 32]. These reductions in BMD have been shown to be further accompanied by increases in BTMs, particularly CTX and PINP, with significantly higher PINP levels observed post-RYGB [33].

In our study, post-RYGB, there were negative associations between VF (intra-, extra-, and total) and both TotalOC and ucOC, suggesting that VF may be linked to reduced osteocalcin levels which may potentially be indicative of impaired bone metabolism following RYGB. Cho et al. [34] reported that higher baseline VF area was associated with greater loss of lumbar BMD after gastrectomy, while higher SF appeared protective against bone loss in patients with non-metastatic gastric cancer [34]. BMD was estimated using computed tomography (CT)-based trabecular attenuation at the first lumbar vertebra, measured in Hounsfield units (HU), with lower values indicating lower BMD [34]. It is known that VF releases proinflammatory cytokines, such as TNF-α and IL-6, which increase bone resorption, decreases BMD, and it was shown that postmenopausal women with obesity who also have visceral adiposity have lower osteocalcin levels [3].

Osteocalcin is the most abundant protein that is secreted by osteoblast and exists in cOC and ucOC forms [35]. While cOC is known to promotes bone mineralization and increases bone strength, ucOC has been shown to play diverse roles, including promoting insulin secretion and β-cell proliferation, stimulating adiponectin release, enhancing insulin sensitivity, reducing fat mass, improving muscle function [35]. We observed negative associations between 2-h post-OGTT glucose levels and TotalOC and ucOC and PINP indicating a potential relationship between bone turnover and glycemic profile. A previous study reported that both ucOC and TotalOC were inversely associated with markers of insulin resistance, such as fasting glucose levels, particularly among individuals without a T2DM diagnosis, while no significant association was found between PINP levels and fasting glucose [36]. TotalOC (cOC and ucOC) is generally considered as a marker of bone turnover [35]. A previous study found a positive association between genetic predisposition to reduced BMD and increased circulating concentrations of TotalOC and cOC [37]. In contrast, no association was observed between genetic risk score and levels of ucOC [37].

In a recent study, it was found that osteocalcin levels were negatively associated with VF and trunk fat, which are indicators of unhealthy fat distribution [38]. Moreover, it has been shown that there is a negative association between serum osteocalcin levels and obesity indices such as BMI, percentage body fat, and waist circumference [39]. It is important to note that 6 months post-MBS, our patients had lost significant amount of their body weight – but they were still considered to be obese with an average BMI of 33 kg/m^2^.

In the SG group, we observed negative association between femoral SF and cOC which may suggest that reductions in femoral SF are associated with changes in bone formation markers and in bone strength. SG procedure has been shown to adversely affect bone health, as indicated by decreases in BMD particularly the femoral neck and total hip, deterioration of bone microarchitecture, increases in marrow adipose tissue, and elevated levels of bone resorption markers [40]. Femoral SF is generally considered to be metabolically protective [8], and Zhang et al. [41] found a positive correlation between thigh fat mass and BMD at both the femoral neck and total lumbar spine. Leg fat mass was also found to be associated with a lower risk of osteoporosis in both postmenopausal women and men with T2DM, independent of total lean mass [42]. Clinically, femoral or thigh SF not only provides mechanical protection to the pelvis and femur [43] but also shows high lipoprotein lipase activity, promoting lipid storage into adipocytes and stimulating new adipocyte formation [44]. The protective lipid storage capacity of femoral SF, measured with CT-radiodensity (HU), remains similar to pre-surgery levels even after significant reduction of fat mass after MBS [45]. This is particularly important as there exist a positive association between dyslipidemia and reduced BMD, particularly among postmenopausal women [46].

The current study has several limitations. The predominance of female participants may limit the generalizability of the findings, as associations between fat distribution and bone metabolism could differ between sexes. While BTMs offer a faster, and potentially more sensitive method to detect dynamic changes in osteoblast and osteoclast activity compared to imaging techniques, they lack the ability to measure changes in bone size, shape, and BMD and mostly reflect the metabolically active trabecular compartment [16]. Additionally, the study’s subjects comprised a heterogeneous group, including subjects with obesity with IFG, IGT, and T2DM, both before and after weight loss through MBS, as well as metabolically healthy individuals without obesity. To minimize the impact of circadian variation in BTM levels, all samples were obtained during a consistent time window (between 08:00 and 10:00 AM) [47]. Another limitation is that our analysis did not investigate the potential influence of serum 25-hydroxyvitamin D3 and intact parathyroid hormone (PTH) levels on the observed associations between serum cytokines and BTMs. In Chinese postmenopausal women with osteopenia and osteoporosis, higher serum levels of 25-hydroxyvitamin are associated with lower levels of BTMs - specifically β-CTX, osteocalcin, and PTH [48]. These associations persist even after adjusting for age and BMI [48], indicating that sufficient vitamin D status may contribute to reduced bone turnover and improved bone health in this population.

In conclusion, this study adds to our growing understanding of the complex relationships between regional fat depots and bone metabolism in the context of obesity and MBS. We found that VF depots were negatively associated with BTMs after RYGB. Femoral SF showed an association with cOC after SG, suggesting potential depot-specific effects of fat loss on bone formation. These findings show the importance of considering fat distribution and surgery types when evaluating bone health outcomes post-MBS and also highlights the need for continued monitoring of skeletal health in this population.

## Supplementary information

Supplemental Fig. 1: Correlation of 2-h glucose and several markers of bone turnover

Supplemental Table 1. Correlations of adipokines and cytokines and bone turnover markers in the study participants

Supplemental Fig. 1

## Data Availability

The datasets generated and/or analysed during the current study are available from the corresponding author on reasonable request.
