# Supplementary material for: Differential associations of subcutaneous and visceral fat with bone turnover markers: A study on bariatric surgery patients with severe obesity and individuals without obesity
Source: Int J Obes (Lond). 2025 Aug 26;49(12):2494–502. doi: 10.1038/s41366-025-01888-1 (PMC12634438; doi:10.1038/s41366-025-01888-1)
Supplement: Supplementary file 1 — Supplemental Fig. 1: Correlation of 2-h glucose and several markers of bone turnover [file 41366_2025_1888_MOESM1_ESM.docx]

**Dadson et al.** Differential Associations of Subcutaneous and Visceral Fat with Bone Turnover Markers: A Study on Bariatric Surgery Patients with Severe Obesity and Individuals Without Obesity

**Supplemental Figure 1**: An inverse correlation was depicted between plasma glucose levels following a 2-hour oral glucose tolerance test (2-hour glucose) and several markers of bone turnover, including C-terminal telopeptide of type 1 collagen (CTX) (r = -0.37, FDR = 0.008) [A], total osteocalcin (TotalOC) (r = -0.37, FDR = 0.004) [B], undercarboxylated osteocalcin (ucOC) (r = -0.32, FDR = 0.02) [C], and serum procollagen type I N-propeptide (PINP) (r = -0.33, FDR = 0.02) [D]. Statistically significant correlations were identified using a Spearman's rank correlation test with an FDR < 0.05. Points were colored to differentiate between patients with severe obesity before (BLUE), and after (RED) metabolic bariatric surgery, and control subjects without obesity (BLACK). Dashed lines indicate 95% confidence intervals for the regression lines.
