# Supplementary material for: Differential associations of subcutaneous and visceral fat with bone turnover markers: A study on bariatric surgery patients with severe obesity and individuals without obesity
Source: Int J Obes (Lond). 2025 Aug 26;49(12):2494–502. doi: 10.1038/s41366-025-01888-1 (PMC12634438; doi:10.1038/s41366-025-01888-1)
Supplement: Supplementary file 2 — Supplemental Table 1. Correlations of adipokines and cytokines and bone turnover markers in the study participants [file 41366_2025_1888_MOESM2_ESM.docx]

|  | TRACP5a | TRACP5b | CTX | PINP | TotalOC | cOC | ucOC |
| --- | --- | --- | --- | --- | --- | --- | --- |
| IL-6 | -0.0001 | -0.08 | -0.10 | -0.02 | -0.04 | -0.04 | -0.06 |
| IL-8 | **0.31*** | 0.07 | -0.04 | -0.07 | -0.09 | -0.23 | 0.12 |
| TNF-α | **0.30**** | 0.04 | -0.08 | -0.09 | -0.12 | -0.23 | 0.01 |
| MCP-1 | 0.13 | 0.04 | -0.08 | -0.07 | -0.12 | -**0.29*** | 0.22 |
| Leptin | 0.04 | -0.17 | **-0.31*** | -0.22 | **-0.32*** | **-0.36**** | -0.08 |
| Resistin | -0.0003 | 0.14 | 0.11 | 0.11 | 0.08 | 0.14 | 0.02 |
| Adiponectin | 0.166 | 0.07 | 0.05 | 0.02 | 0.12 | 0.02 | 0.14 |

Supplementary table 1. Correlations of adipokines and cytokines and bone turnover markers in the study participants

The data presented here are Pearson correlation coefficients of cytokines and bone turnover markers, including Tartrate-resistant acid phosphatase 5a/b (TRACP5a/b); C-terminal telopeptide of type I collagen (CTX); procollagen type I N-terminal propeptide (PINP); total (TotalOC), carboxylated (cOC), and undercarboxylated (ucOC) osteocalcin; interleukin-6/8 (IL-6/8); tumor necrosis factor-alpha (TNF-α); monocyte chemoattractant protein-1 (MCP-1). FDR-adjusted significance levels are indicated as *p < 0.05, **p < 0.01, and ***p < 0.001 for the study participants. Statistically significant results (p < 0.05) are highlighted in bold.
