# Supplementary figures and images for: Differential associations of subcutaneous and visceral fat with bone turnover markers: A study on bariatric surgery patients with severe obesity and individuals without obesity
Source: Int J Obes (Lond). 2025 Aug 26;49(12):2494–502. doi: 10.1038/s41366-025-01888-1 (PMC12634438; doi:10.1038/s41366-025-01888-1)

**A**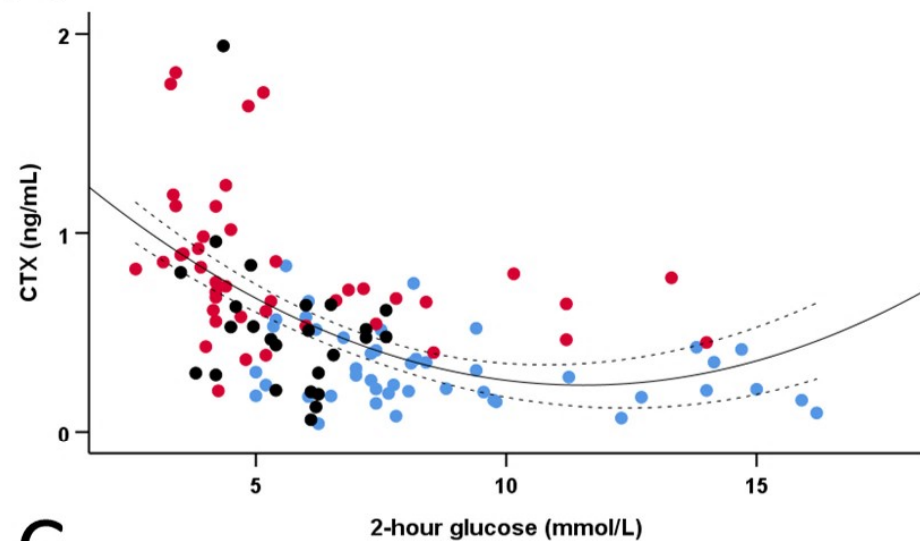**B**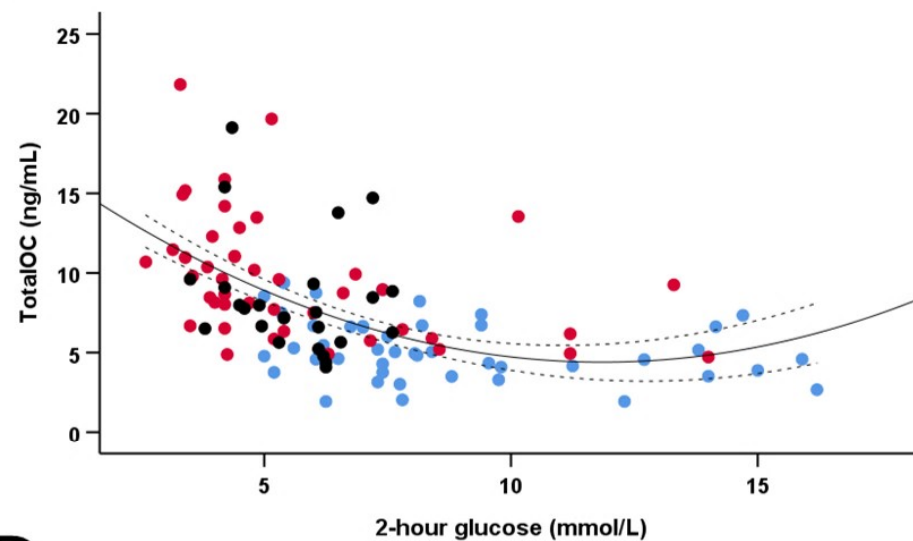**C**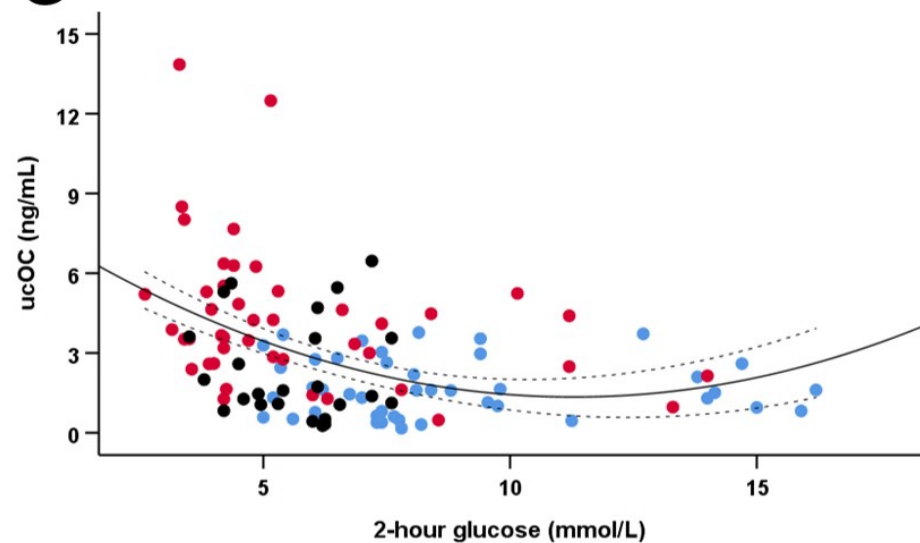**D**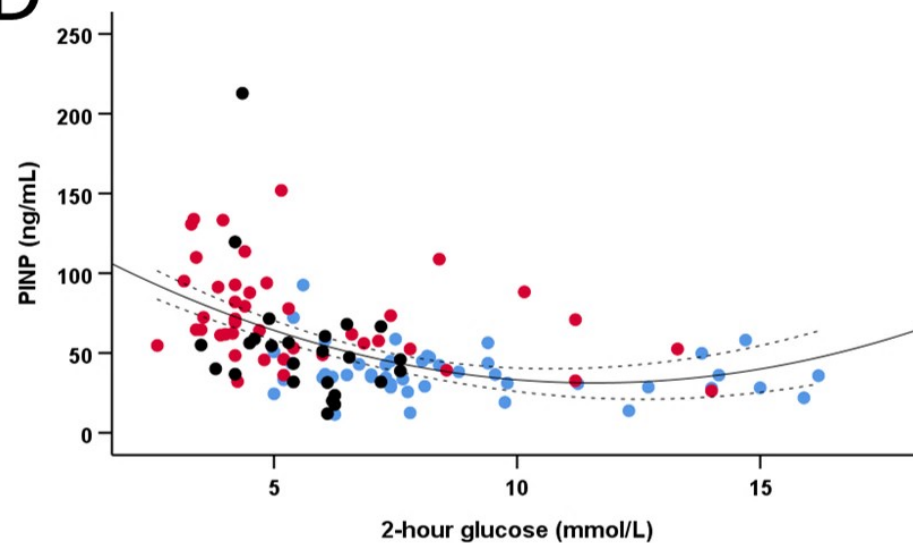

Supplement: Supplementary file 3 — Supplemental Fig. 1 [file 41366_2025_1888_MOESM3_ESM.pdf]
